# A Sub-0.01 °C Resolution All-CMOS Temperature Sensor with 0.43 °C/−0.38 °C Inaccuracy and 1.9 pJ · K^2^ Resolution FoM for IoT Applications

**DOI:** 10.3390/mi15091132

**Published:** 2024-09-06

**Authors:** Yixiao Sun, Jie Cheng, Zhizhong Luo, Yanhan Zeng

**Affiliations:** 1 School of Electronics and Communication Engineering, Guangzhou University, Guangzhou 510006, China; analog_syx@163.com (Y.S.); 2019500007@e.gzhu.edu.cn (J.C.); 18129536621@163.com (Z.L.); 2 Key Lab of Si-based Information Materials & Devices and Integrated Circuits Design, Guangzhou University, Guangzhou 510006, China

**Keywords:** time-domain temperature sensor, high resolution, subthreshold, VTC, ROSC, time-to-digital converter (TDC)

## Abstract

A high resolution, acceptable accuracy and low power consumption time-domain temperature sensor is proposed and simulated in this paper based on a 180 nm standard CMOS technology. A diode stacking structure is introduced to enhance the accuracy of the temperature sensing core. To improve the resolution of the sensor, a dual-input capacitor multiplexing voltage-to-time converter (VTC) is implemented. Additionally, a low-temperature drift voltage-mode relaxation oscillator (ROSC) is proposed, effectively reducing the large oscillation frequency drift caused by significant temperature impacts on delay errors. The simulated results show that the resolution is as high as 0.0071 °C over 0∼120 °C with +0.43 °C/−0.38 °C inaccuracy and 1.9 pJ · K^2^ resolution FoM, consuming only 1.48 μW at a 1.2 V supply voltage.

## 1. Introduction

The Internet of Things (IoT) is rapidly expanding, incorporating smart wearables and intelligent household appliances that enhance daily convenience [1]. As a result, there is a growing demand for temperature sensors in these applications, necessitating features like low power consumption, compact size, high accuracy, and especially excellent resolution [2,3,4]. CMOS temperature sensors, which are preferred for their energy efficiency and ease of integration with other signal-processing units, effectively meet these requirements [5,6,7]. They support on-chip dynamic thermal management across various IoT devices, ensuring efficient heat dissipation and optimal performance.

CMOS temperature sensors typically require a high-quality frequency reference circuit to convert temperature-related information such as delay/frequency into digital codes [8]. Using an off-chip reference poses challenges in system integration and miniaturization while integrating an on-chip frequency reference can significantly reduce the overall size and power consumption of the sensor chip [9]. Therefore, a fully integrated reference circuit and clock generator are often considered essential in CMOS temperature sensors.

The existing classification of CMOS temperature sensors is based on the types of temperature-sensing devices used in CMOS processes, namely BJT-based, resistive-based, all-MOSFET-based, and TD-based [10]. For example, in a BJT-based front-end circuit, the temperature-related signal it generates is an analog voltage signal, specifically $V_{BE}$ and $\Delta V_{BE}$ [11]. However, there are various ways to digitize this signal. For instance, the temperature-related voltage can be directly quantified using an analog-to-digital converter (ADC), or it can be first converted into a current, and then a current-mode ADC can be designed to quantify this current. Alternatively, the temperature-related voltage or current can be converted into delay, frequency, or other signals that can be directly processed by digital circuits. Based on the different methods of converting analog signals into digital signals, common CMOS temperature sensor architectures can be categorized into four domains: the voltage domain, current domain, frequency domain, and time domain.

**Figure 1:**
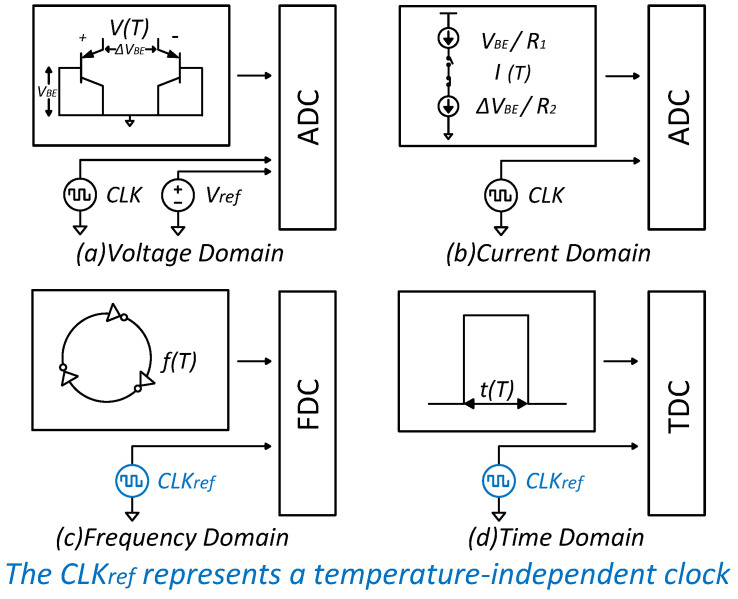
Operation principle of four typical temperature sensors.

Figure 1 displays the basic diagrams of the four typical temperature sensors reported above. It is important to note that the $CLK_{ref}$ before FDC and TDC represents a temperature-independent clock.

As shown in Figure 1a, BJT-based voltage-domain temperature sensors are capable of high precision utilizing a SAR ADC or a ΔΣ ADC, enhanced by various calibration techniques [12]. The precision of these sensors is minimally affected by small variations in clock frequency, so they typically do not require a temperature-independent clock signal. However, voltage-domain CMOS temperature sensors are not limited to BJT-based temperature sensors. By integrating with other types of temperature-sensing devices and custom ADCs, different performance requirements can be met [13].

For example, in BJT-based temperature sensors, the current-domain solution modifies the analog front-end circuit to convert $\Delta V_{BE}$ and $V_{BE}$ into temperature-related currents. These currents are then quantified using a current-domain ΔΣ modulator, as illustrated in Figure 1b [14]. Compared to direct voltage readout, current-domain readout reduces dependence on voltage swing and, in low-precision scenarios, it offers advantages such as smaller areas and lower voltage supply. Similar to the voltage-domain solution, if combined with different types of temperature-sensing devices, the current-domain temperature sensor can also achieve a wider range of performance options [15].

Time-domain CMOS temperature sensors mainly adopt two approaches. One approach outputs duty cycle information proportional to temperature, producing a duty cycle-modulated square wave (or PWM wave) that can be directly processed by digital circuits, simplifying the circuit interface [16]. The other type, shown in Figure 1d, directly converts temperature into temperature-related pulse signals or periodic time-domain signals, which are subsequently quantified using an all-digital TDC [17]. However, this approach requires an additional precise clock as a reference signal for the TDC to digitize the temperature-related time-domain signal. Compared to traditional voltage-domain or current-domain readouts, TDCs used in time-domain readouts are better suited to digital circuit implementation, allowing their area and power consumption to benefit from process node scaling.

As shown in Figure 1c, similar to the time-domain readout architecture, converting temperature into frequency signals is another viable approach. Like the time-domain method, the frequency-domain approach converts temperature into delay information; however, instead of directly quantifying the width of a single pulse, it measures the signal frequency generated by the sensor. This significantly reduces the need for a single delay duration, enabling a smaller area and faster temperature measurement, although the temperature measurement error is typically larger [18].

In this paper, a CMOS-based time-domain temperature sensor suitable for IoT applications is proposed. To improve accuracy in the temperature sensing core, a diode stacking structure is implemented, generating $V_{PTAT}$ and $V_{CTAT}$ with high-temperature coefficients (TCs). A dual-input capacitor multiplexing voltage-to-time converter is introduced to enhance resolution by fully utilizing both temperature-negative and temperature-positive correlated information. Furthermore, a low-temperature drift voltage-mode relaxation oscillator is proposed, effectively mitigating large oscillation frequency drift due to temperature-induced delay errors.

The remainder of this paper is organized as follows. Section 2 describes the system architecture and operation principle of the proposed temperature sensor. Section 3 shows the circuit implementation details of the three main parts. Section 4 demonstrates the simulation results with comparison to recently reported temperature sensors, while Section 5 concludes this paper.

## 2. Architecture and Operation Principle

**Figure 2:**
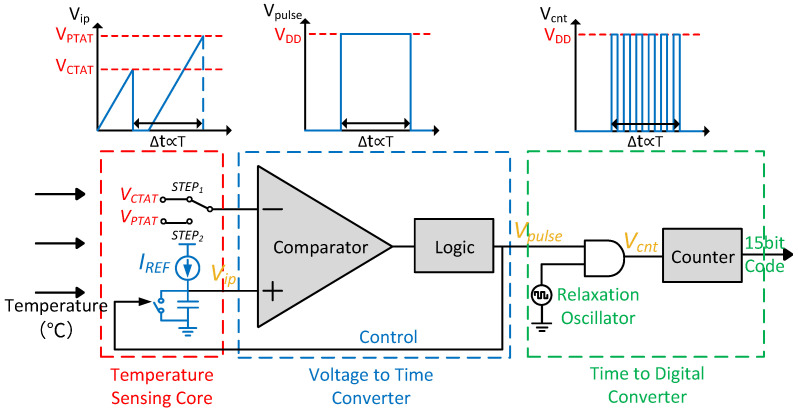
The structural diagram of the proposed temperature sensor.

Figure 2 shows the block diagram of the proposed temperature sensor. The structure consists of three major parts: (1) a temperature sensing core, (2) a voltage-to-time converter, and (3) a time-to-digital converter with a temperature-stabilized relaxation oscillator.

The proposed temperature sensing core serves the purpose of generating temperature-related signals with high TC ($V_{PTAT}$/$V_{CTAT}$) and reference signals ($I_{REF}$/$V_{REF}$). The relaxation oscillator generates the reference frequency ($f_{REF}$) using $V_{REF}$. The dual-input capacitor multiplexing voltage-to-time converter is proposed to convert $V_{PTAT}$ and $V_{CTAT}$ to temperature-dependent time quantity Δt, as represented by the width of $V_{pulse}$. Since Δt is proportional to temperature, digitization is performed by converting Δt to 15 bits of digital code via a proposed time-to-digital converter clocked by an on-chip relaxation oscillator. To ensure digitization is not influenced by temperature, the oscillator is designed to be temperature stable [19].

**Figure 3:**
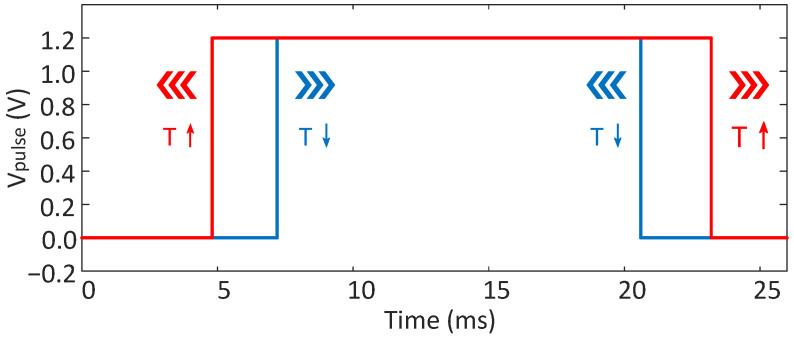
The time signal $V_{pulse}$ as temperature increases and decreases.

As shown in Figure 2, the capacitor is successively charged to $V_{PTAT}$ and $V_{CTAT}$ by $I_{REF}$ [20]. During the time from when $V_{ip}$ charges to $V_{CTAT}$ until it charges to $V_{PTAT}$, the logic circuit generates the time signal $V_{pulse}$. As the temperature rises, the time required to charge to $V_{CTAT}$ shortens, while the time to charge to $V_{PTAT}$ lengthens. With the combination of high-TC CTAT and PTAT signals, the temperature-dependent time quantity Δt is significantly extended with temperature, thus improving the resolution [21]. The change in the time signal $V_{pulse}$ as the temperature increases and decreases is shown in Figure 3.

To support the above point, assuming there are two temperature points $T_{1}$ and $T_{2}$ ($T_{1}$>$T_{2}$), the change in Δt as the temperature increases can be represented as follows:

(1) $$\begin{array}{cc} \Delta t=\Delta t\left(T_{1}\right)-\Delta t\left(T_{2}\right) & =\frac{C\left[k_{PTAT}\left(T_{1}-T_{2}\right)+k_{CTAT}\left(T_{2}-T_{1}\right)\right]}{I_{REF}}\\ \; & =\frac{C\left(k_{PTAT}-k_{CTAT}\right)\left(T_{1}-T_{2}\right)}{I_{REF}}, \end{array}$$

where $k_{CTAT}$ and $k_{PTAT}$ are the absolute value of the TC of $V_{CTAT}$ and $V_{PTAT}$, respectively, while C represents the capacitance in Figure 2.

As a supplement, in Equation (1), we assume that $V_{CTAT}$ and $V_{PTAT}$ exhibit ideal linear temperature characteristics, considering only first-order temperature dependency, with minimal influence from higher-order terms.

## 3. Circuit Implementation Details

This section provides a detailed overview of the three key components mentioned earlier, focusing on the innovative strategies used to enhance the core performance of the temperature sensor and highlighting the unique contributions of our work.

### 3.1. Temperature Sensing Core

**Figure 4:**
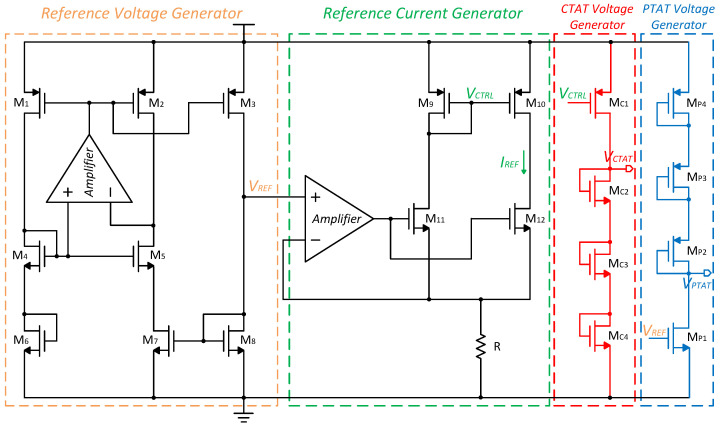
The schematic of the proposed temperature sensing core.

The proposed temperature sensing core is shown in Figure 4. The current negative feedback of the amplifier is used to increase the output impedance so that the output current is weakly correlated with the supply voltage. $V_{REF}$ is the reference voltage provided by the pre-stage reference circuit, with a resistor R composed of positive and negative temperature coefficient resistors, ensuring the output current is independent of temperature. The reference current and reference voltage in the initial part are applied in the implementation of PTAT and CTAT voltages, while the diode-stacked architecture achieves temperature-dependent voltages with larger temperature coefficients.

The schematic of the $V_{REF}$ generator is shown in the yellow dotted box [22]. $M_{4}$ and $M_{5}$ operate in the subthreshold region, $M_{6}$ and $M_{7}$ operate in the linear triode region, and other transistors operate in the saturation region. M6 is used to reduce power consumption by reducing the static current of the branch. $M_{2}$ and $M_{3}$ are the same, so the current of $M_{7}$ is equal to the current of $M_{8}$. Therefore,

(2) $$\begin{array}{c} \mu C_{ox}S_{7}\left[\left(V_{REF}-V_{TH7}\right)V_{DS7}-\frac{1}{2}{V_{DS7}}^{2}\right]=\frac{1}{2}\mu C_{ox}S_{8}{\left(V_{REF}-V_{TH8}\right)}^{2}, \end{array}$$

where μ is the carrier mobility and $C_{ox}$ is the gate-to-oxide capacitance, *S* is the width-to-length ratio *W*/*L* of the transistor, $V_{TH}$ is the threshold voltage of the transistor, and $V_{DS}$ is the drain-source voltage of the transistor.

In the same process, the threshold voltage of the MOS transistor is nearly equal under the same type, so $V_{TH7}$≈$V_{TH8}$; therefore,

(3) $$\begin{array}{c} V_{REF}=V_{TH}+\eta \frac{kT}{q}\ln\left(\frac{S_{5}}{S_{4}}\right)\left[\frac{S_{7}}{S_{8}}+\sqrt{{\left(\frac{S_{7}}{S_{8}}\right)}^{2}-\frac{S_{7}}{S_{8}}}\right]. \end{array}$$

The relationship between threshold voltage and temperature is given by the following:

(4) $$\begin{array}{c} V_{TH}\left(T\right)=V_{TH0}-\frac{dV_{TH}}{dT}T, \end{array}$$

where $V_{TH0}$ is the threshold voltage of the transistor at 0 K, and $\frac{dV_{TH}}{dT}$ is the temperature coefficient of the threshold voltage. According to Equations (3) and (4), as long as

(5) $$\begin{array}{c} \frac{dV_{TH}}{dT}=\eta \frac{k}{q}\ln\left(\frac{S_{5}}{S_{4}}\right)\left[\frac{S_{7}}{S_{8}}+\sqrt{{\left(\frac{S_{7}}{S_{8}}\right)}^{2}-\frac{S_{7}}{S_{8}}}\right], \end{array}$$

a reference voltage $V_{REF}$ independent of temperature can be obtained, thereby further achieving the reference current.

The CTAT and PTAT voltage generators are biased through the $I_{REF}$ and $V_{REF}$. It is noteworthy that all MOSFETs of the CTAT and PTAT voltage generator operate in the subthreshold region, as shown in the red dotted box and blue dotted box, respectively. The subthreshold leakage current of the MOS transistor is exponentially related to the gate and drain voltages. The I-V characteristics can be expressed as follows:

(6) $$\begin{array}{c} I_{D}=\mu C_{ox}S\left(\eta -1\right)V_{T}^{2}\exp\left(\frac{V_{GS}-V_{TH}}{\eta V_{T}}\right)\left[1-\exp\left(-\frac{V_{DS}}{V_{T}}\right)\right], \end{array}$$

where η is the subthreshold slope factor, $V_{GS}$ is the gate-source voltage of the transistor, and $V_{T}$ is the thermal voltage ($V_{T}$ = kT/*q*, where *k* is the Boltzmann constant, *q* is the electron charge, and *T* is the temperature).

For typical application temperature ranges, it holds that $V_{DS}$≫ 4 $V_{T}$, and we can ignore the effect of $V_{DS}$ on the leakage current $I_{D}$. Consequently, at this time, $I_{D}$ can be approximated as follows:

(7) $$\begin{array}{c} I_{D}\approx \mu C_{ox}S\left(\eta -1\right)V_{T}^{2}\exp\left(\frac{V_{GS}-V_{TH}}{\eta V_{T}}\right). \end{array}$$

Therefore, the expression for $V_{GS}$ can be represented by the following equation:

(8) $$\begin{array}{c} V_{GS}=V_{TH}+\eta V_{T}\ln\left(M\right), \end{array}$$

(9) $$\begin{array}{c} M=\frac{I_{D}}{\mu C_{ox}S\left(\eta -1\right)V_{T}^{2}}, \end{array}$$

(10) $$\begin{array}{c} \mu =\mu_{0}{\left(\frac{T}{T_{0}}\right)}^{-m}, \end{array}$$

where $\mu_{0}$ is the mobility at $T_{0}$ and *m* is the mobility temperature exponent. The value of m is about 1.5 in standard CMOS technologies according to [23], so the temperature coefficient in the logarithmic term can be disregarded. By taking the partial derivative of Equation (8), the TC of $V_{GS}$ can be expressed as follows:

(11) $$\begin{array}{c} \frac{\partial V_{GS}}{\partial T}=\frac{\partial V_{TH}}{\partial T}+\eta \frac{k_{B}}{q}\ln\left(M\right). \end{array}$$

Based on the fact that $\left|\frac{\partial V_{TH}}{\partial T}\right|>\eta \frac{k_{B}}{q}\ln\left(M\right)$, it follows that $\frac{\partial V_{GS}}{\partial T}<0$, leading to a negative temperature coefficient (NTC) of $V_{GS}$. Drawing from the earlier discussions, as illustrated in Figure 4, the utilization of the diode stacking structure serves to treble the TC of $V_{GS}$ and generate $V_{CTAT}$:

(12) $$\begin{array}{cc} \frac{\partial V_{CTAT}}{\partial T} & =\frac{\partial V_{GSC2}}{\partial T}+\frac{\partial V_{GSC3}}{\partial T}+\frac{\partial V_{GSC4}}{\partial T}\\ \; & =\frac{\partial V_{THC2}}{\partial T}+\frac{\partial V_{THC3}}{\partial T}+\frac{\partial V_{THC4}}{\partial T}+\eta \frac{k}{q}\ln\left(M_{C2}M_{C3}M_{C4}\right). \end{array}$$

Furthermore, the $V_{PTAT}$ is formed using a principle similar to that of $V_{CTAT}$:

(13) $$\begin{array}{cc} V_{PTAT} & =V_{DSP1}=V_{DD}-V_{SGP2}-V_{SGP3}-V_{SGP4}. \end{array}$$

Due to the NTC of $V_{GSP2}$, $V_{GSP3}$, and $V_{GSP4}$, $V_{PTAT}$ exhibits a positive temperature coefficient (PTC). Additionally, the utilization of the diode stacking technique serves to treble the TC of $V_{PTAT}$. The high TC voltages obtained above will be beneficial for improving the overall resolution.

### 3.2. Voltage-to-Time Converter

**Figure 5:**
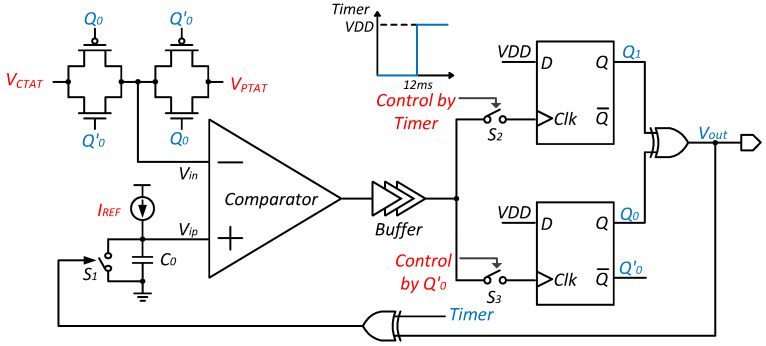
The schematic of the proposed VTC.

The logic circuit of the proposed dual-input capacitor multiplexing VTC is shown in Figure 5. The TIMER signal is used for time-setting purposes, controlling switch $S_{2}$ and the XOR gate that, in turn, controls switch $S_{1}$. It generates a rising edge at 12 ms, after $V_{ip}$ equals $V_{CTAT}$, to ensure the capacitor is fully discharged and to prevent overlapping the first and second charging processes. The two TG gates, controlled by signals $Q_{0}$ and $Q_{0}^{\prime }$, sequentially connect the inverting input of the comparator, $V_{in}$, to $V_{CTAT}$ and $V_{PTAT}$. Switch $S_{3}$ is also controlled by $Q_{0}^{\prime }$. The output signals $Q_{0}$ and $Q_{1}$ from the two D flip-flops are XORed to produce $V_{out}$, which is then XORed again with the TIMER signal to control switch $S_{1}$. In the initial state, the D flip-flops are reset, setting both $Q_{0}$ and $Q_{1}$ to 0.

**Figure 6:**
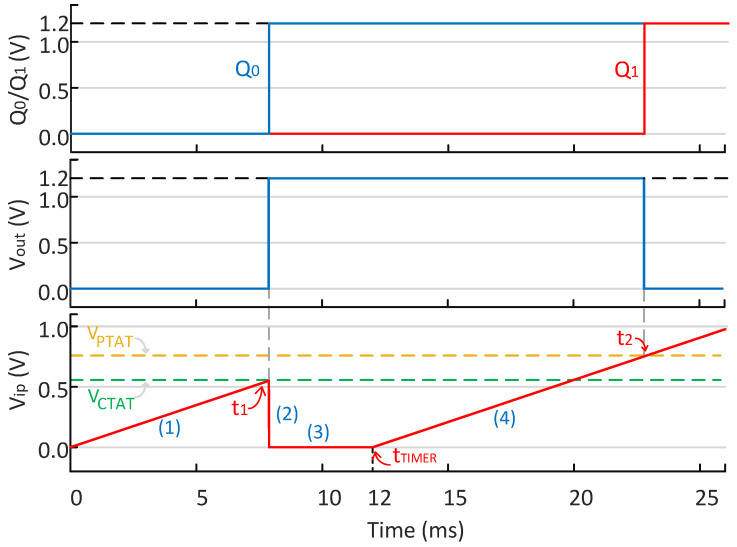
The change in signals $V_{ip}$ and $V_{out}$ over time.

The operation process can be divided into four steps as shown in Figure 6.

(1) First charging: The $C_{0}$ is charged to $V_{CTAT}$ by $I_{REF}$. During this period, $Q_{0}^{\prime }$ is at a high level; thus, switch $S_{3}$ is turned off, and $V_{CTAT}$ is connected to the inverting input of the comparator, while $V_{out}$ remains at a low level.

(2) Discharging: When switch $S_{1}$ is closed, $V_{ip}$ is discharged to zero after the $V_{ip}$ surpasses $V_{CTAT}$ and $V_{out}$ rises. At this time, $Q_{0}$ reaches a high level, and $V_{PTAT}$ is connected to the inverting input of the comparator.

(3) Time slot: This period allows sufficient time to ensure that the capacitor can fully discharge at any temperature until the TIMER signal appears, moving to the next stage.

(4) Second charging: The $C_{0}$ is charged to $V_{PTAT}$ by $I_{REF}$. During this time, switch $S_{2}$ is closed, while $S_{1}$ and $S_{3}$ are opened. When $V_{ip}$ surpasses $V_{PTAT}$, $Q_{1}$ reaches a high level and $V_{out}$ goes low.

The points in time when $V_{ip}$ reaches $V_{CTAT}$ and $V_{PTAT}$ are denoted as $t_{1}$ and $t_{2}$, respectively, which can be described as follows:

(14) $$\begin{array}{c} t_{1}=\frac{C_{0}V_{CTAT}}{I_{REF}}, \end{array}$$

(15) $$\begin{array}{c} t_{2}=t_{TIMER}+\frac{C_{0}V_{PTAT}}{I_{REF}}. \end{array}$$

As shown in Figure 6, the period Δt during which $V_{out}$ stays high lies exactly between $t_{1}$ and $t_{2}$, which can be expressed as follows:

(16) $$\begin{array}{c} \Delta t=t_{2}-t_{1}=t_{TIMER}+\frac{C_{0}\left(V_{PTAT}-V_{CTAT}\right)}{I_{REF}}. \end{array}$$

As mentioned before, $k_{CTAT}$ and $k_{PTAT}$ are the absolute values of the TC of $V_{CTAT}$ and $V_{PTAT}$. Equation (16) can be rewritten as follows:

(17) $$\begin{array}{c} \Delta t=t_{TIMER}+\frac{C_{0}\left(k_{PTAT}+k_{CTAT}\right)T}{I_{REF}}+Const. \end{array}$$

According to Equation (17), by examining the coefficient of the temperature-related term in Δt, it is evident that the proposed dual-input capacitor multiplexing VTC increases the variation in $V_{out}$’s high-level duration with temperature by fully leveraging the large temperature coefficients of both $V_{CTAT}$ and $V_{PTAT}$ generated by the temperature sensing core. As a result, since the reference frequency provided by the subsequent relaxation oscillator remains constant with temperature, the proposed VTC significantly enhances resolution.

**Figure 7:**
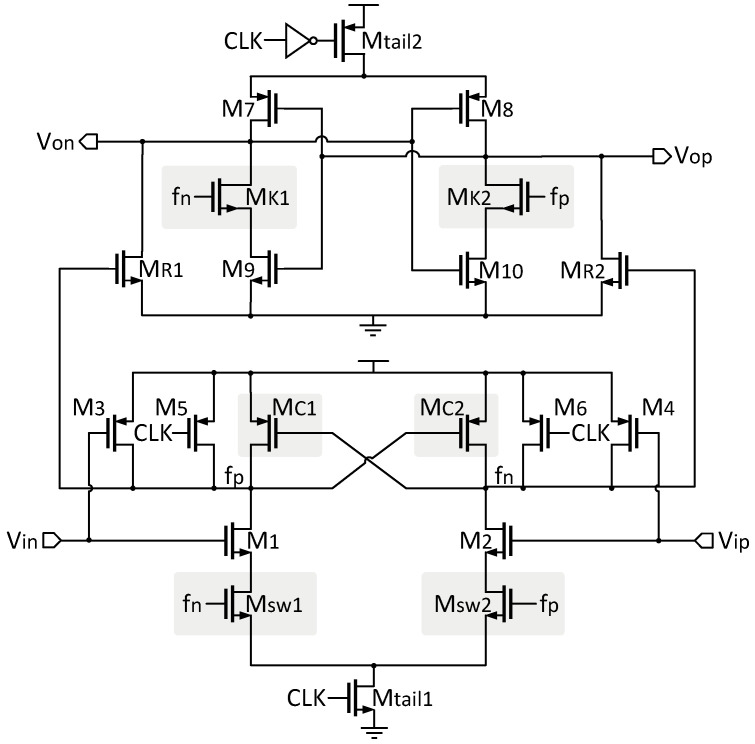
The comparator in the proposed VTC.

The comparator in the proposed VTC is shown in Figure 7. The structure is modified from the traditional double-tail comparator by introducing a cross-coupled configuration to increase the latch’s regeneration speed under low-power constraints [24]. This modification significantly reduces the delay time and avoids unnecessary errors during the comparison of $V_{in}$ and $V_{ip}$ in the VTC.

The comparator delay, $t_{delay}$, consists of two parts: $t_{0}$ and $t_{latch}$. The delay $t_{0}$ represents the charging time of the load capacitance $C_{Lout}$ at the output nodes $V_{on}$ and $V_{op}$ of the latch stage. The delay $t_{latch}$ represents the latch’s regeneration time, which is related to the initial output voltage difference $\Delta V_{0}$ and the effective transconductance $g_{meff}$ of the latch. Specifically, the larger the $\Delta V_{0}$ and $g_{meff}$, the shorter the regeneration time.

For the proposed dynamic comparator based on a cross-coupled structure, two control transistors, $M_{C1}$ and $M_{C2}$, are added to the pre-amplification stage in parallel with the $M_{5}$/$M_{6}$ transistors but in a cross-coupled manner to increase the differential voltage $\Delta V_{fn/fp}$ between $f_{n}$ and $f_{p}$. This, in turn, increases the output voltage difference $\Delta V_{0}$ at the start of latch regeneration. By adding two key transistors, $M_{K1}$ and $M_{K2}$, to the latch stage, the effective transconductance of the intermediate stage transistors is increased. The cross-coupled structure causes one of the first-stage output nodes ($f_{n}$/$f_{p}$) to be charged back to $V_{DD}$ at the beginning of the decision phase, turning on one of the intermediate stage transistors and thereby increasing the latch’s effective transconductance $g_{meff}$. In other words, positive feedback is enhanced [24].

In summary, the increases in $\Delta V_{0}$ and $g_{meff}$ improve the latch regeneration speed, reducing the overall comparator delay $t_{delay}$, which perfectly meets the requirements of the proposed VTC.

Additionally, it is worth noting that the pre-amplification stage introduces NMOS switch transistors $M_{sw1}$ and $M_{sw2}$, controlled by $f_{n}$ and $f_{p}$, respectively. These transistors simulate the latch operation, minimizing static power consumption and ensuring the low power consumption of the comparator circuit.

### 3.3. Relaxation Oscillator

**Figure 8:**
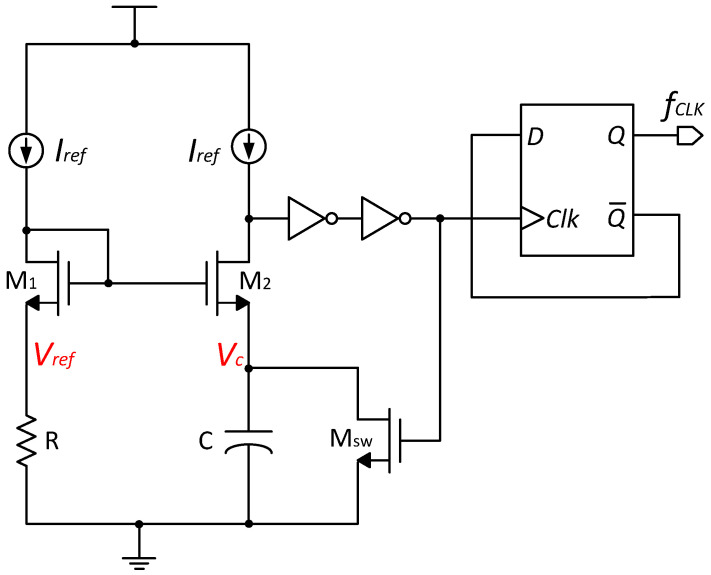
The schematic of a conventional ROSC.

The conventional current-mode relaxation oscillator is shown in Figure 8. In this design, the current in the $M_{1}$ branch generates the voltage reference $V_{ref}$, which is replicated to the $M_{2}$ branch to charge the capacitor C. When the voltage $V_{c}$ on capacitor C exceeds $V_{ref}$ across resistor R, the buffer generates a pulse that both switches the circuit to discharge the capacitor and feeds into the clock divider to produce the output clock. After a time τ, the transistor $M_{SW}$ is turned on, discharging the capacitor C and completing the oscillation cycle [25]. This current-mode relaxation oscillator eliminates the need for comparators, significantly reducing power consumption.

In summary, the output frequency of the relaxation oscillator is determined by the inverter delay and the charge/discharge time of the capacitor, which are in turn determined by the reference current $I_{ref}$. The oscillation period $T_{RC}$ is given by the following:

(18) $$\begin{array}{c} T_{RC}=\frac{V_{ref}C}{I_{ref}}+\tau =RC+\tau . \end{array}$$

Using a D flip-flop for frequency division, the output frequency $f_{CLK}$ of the relaxation oscillator is as follows:

(19) $$\begin{array}{c} f_{CLK}=\frac{1}{2T_{RC}}=\frac{1}{2(RC+\tau )}. \end{array}$$

Typically, conventional current references have a nonlinear temperature coefficient, making it difficult to compensate for the temperature dependency of the output frequency. To achieve weak temperature dependence of frequency, traditional methods often use series and parallel combinations of resistors with positive and negative temperature coefficients [25]. However, the accuracy achieved by these methods is insufficient to meet the stringent requirements of temperature sensors, and it is generally challenging to achieve a frequency with a minimal temperature drift at higher oscillation frequencies.

**Figure 9:**
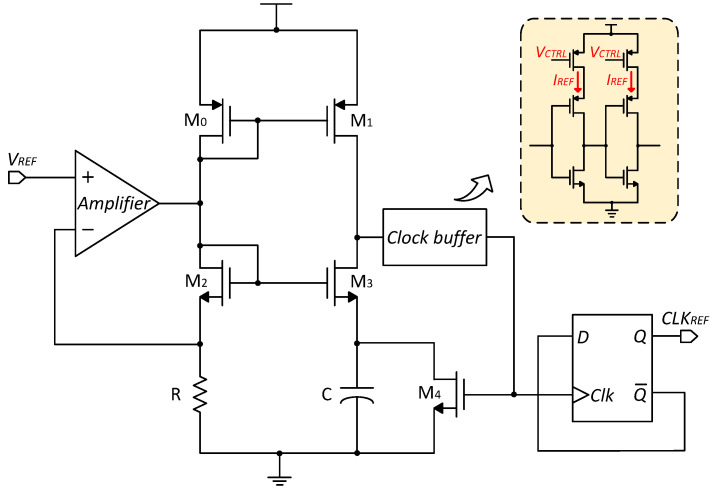
The schematic of the proposed ROSC.

Figure 9 shows the schematic of the proposed voltage-mode relaxation oscillator. The amplifier ensures that the source voltage of $M_{2}$ equals $V_{REF}$, which has a low-temperature dependency. This voltage, together with resistor R, sets the current in the $M_{2}$ branch, which is then mirrored to the $M_{3}$ branch. The resistor R is composed of two resistors, $R_{a}$ and $R_{b}$: $R_{a}$ is an n-well resistor with a positive temperature coefficient, while $R_{b}$ is a poly resistor with a negative temperature coefficient. By adjusting the ratio of these two resistors, a temperature-independent equivalent resistance can be achieved, resulting in an output frequency with minimal temperature dependence.

In addition, the equation for the transmission delay of the inverter is as follows:

(20) $$\begin{array}{c} \tau_{inv}=0.69C_{L}\frac{R_{eqn}+R_{eqp}}{2}. \end{array}$$

(21) $$\begin{array}{c} R_{eq}=\frac{3V_{DD}}{4I_{0}}\left(1-\frac{7}{9}\lambda V_{DD}\right). \end{array}$$

Therefore,

(22) $$\begin{array}{c} \tau \propto \frac{V_{DD}C_{L}}{I_{inv}}. \end{array}$$

$R_{eq}$ is the equivalent on-resistance of the inverter, $C_{L}$ is the load capacitance, $I_{0}$ is the speed saturation current of the inverter, and $I_{inv}$ is the buffer bias current.

From Equation (18), the oscillation frequency depends on three parameters: R, C, and τ. Since R and C are independent of temperature, minimizing the variation in τ can improve the accuracy of the oscillation frequency. According to Equation (22), τ is inversely proportional to the bias current. Thus, by using a temperature-independent current source $I_{REF}$ to bias the inverter, the oscillator period becomes independent of delay errors and is determined solely by the resistor and capacitor values. This design effectively reduces the impact of delay errors on the oscillation frequency, enhancing the overall stability and accuracy of the oscillator across varying temperatures.

### 3.4. Time-to-Digital Converter

The preceding stage of the TDC, known as a temperature-to-time converter, converts temperature into time. This converter outputs a pulse whose width is ideally linearly proportional to temperature, enabling the conversion of temperature to digital form by digitizing the pulse width. Since the proposed oscillator has a zero temperature coefficient, the temperature-to-digital relationship remains linear, which is crucial for accurate temperature measurement.

**Figure 10:**
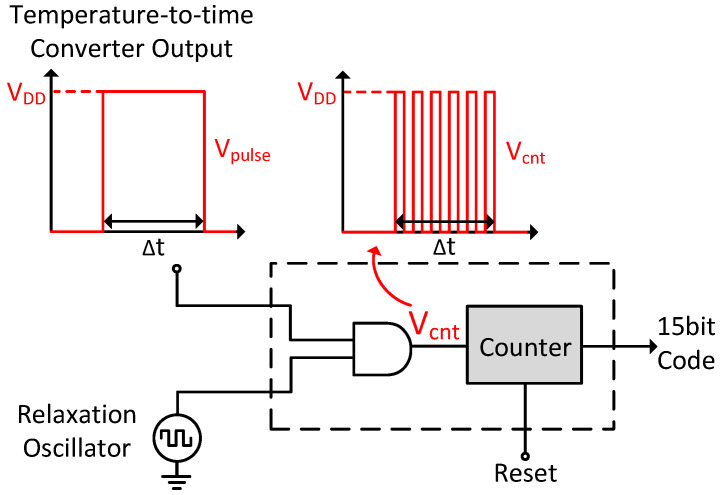
The schematic of the aforementioned TDC.

The block diagram of the TDC is shown in Figure 10. In this configuration, the output from the temperature-to-time converter and a clock signal from an on-chip relaxation oscillator are connected to an AND gate, allowing counting only during the pulse’s high period (Δt) for precise timing measurements. To prevent overflow and ensure accurate counting across the entire temperature range of 0 °C to 120 °C, while accounting for potential offsets due to process variations, a 15-bit asynchronous counter is employed [26].

## 4. Simulation Results and Discussion

The proposed temperature sensor has been implemented using a 180 nm process. The most important parameters for a temperature sensor include resolution, accuracy, and power consumption. In the following sections, we will discuss how such excellent performance is achieved, with a particular focus on the principles behind the ultra-high resolution.

Temperature resolution refers to the smallest temperature change that a temperature sensor can detect, i.e., the smallest temperature variation that can cause a change in the least significant bit (LSB) of the digital output code. Regardless of the type of readout method used in a temperature sensor, the resolution is equal to the ratio of the temperature range to the number of digital codes used for quantization. For the time-domain temperature sensor proposed in this paper, the overall resolution is directly determined by the sensitivity of $V_{out}$ to temperature and the $CLK_{REF}$.

**Figure 11:**
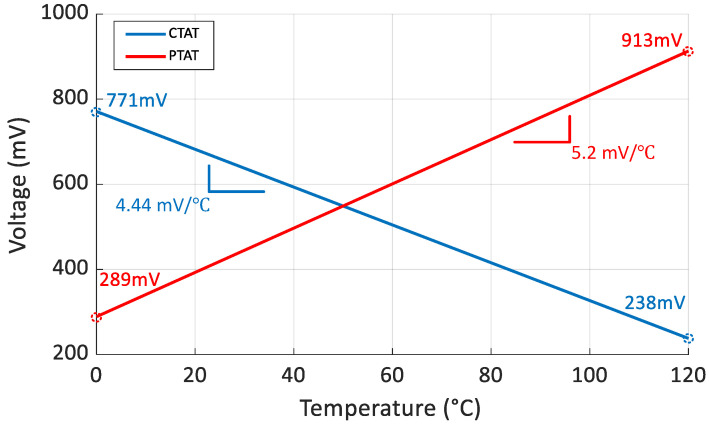
The $V_{PTAT}$ and $V_{CTAT}$ generated by the temperature sensing core.

Firstly, the utilization of a diode stacking structure results in a high slope of 5.2 mV/°C and 4.44 mV/°C for $V_{PTAT}$ and $V_{CTAT}$, respectively, as shown in Figure 11. Next, a dual-input capacitor multiplexing VTC is introduced, amplifying the effects of the high TC $V_{PTAT}$ and $V_{CTAT}$, thereby increasing the sensitivity of $V_{out}$ to temperature.

**Figure 12:**
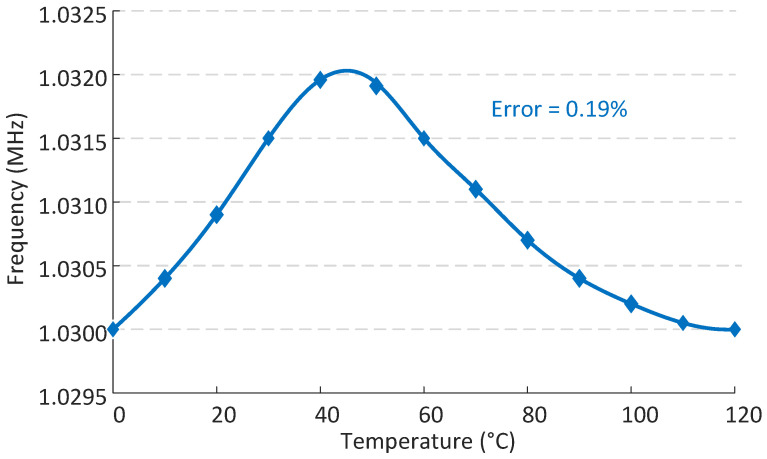
The reference frequency generated by the relaxation oscillator.

It is also noteworthy that the self-designed relaxation oscillator achieved an oscillation frequency higher than 1.03 MHz, as shown in Figure 12. Ultimately, an excellent resolution of 0.0071 °C was achieved.

The temperature sensor’s accuracy is primarily dependent on the precision of the reference frequency and the stability of the temperature coefficients of $V_{PTAT}$ and $V_{CTAT}$. Figure 12 presents the variation in $f_{REF}$, achieving a variation of as low as 0.19 %. The previously mentioned stability can be represented by the TC of $\frac{\partial V_{PTAT}}{\partial T}$ and $\frac{\partial V_{CTAT}}{\partial T}$. Within the temperature range of 0∼120 °C, the simulated values for these coefficients are 113 ppm/°C and 105 ppm/°C, respectively.

**Figure 13:**
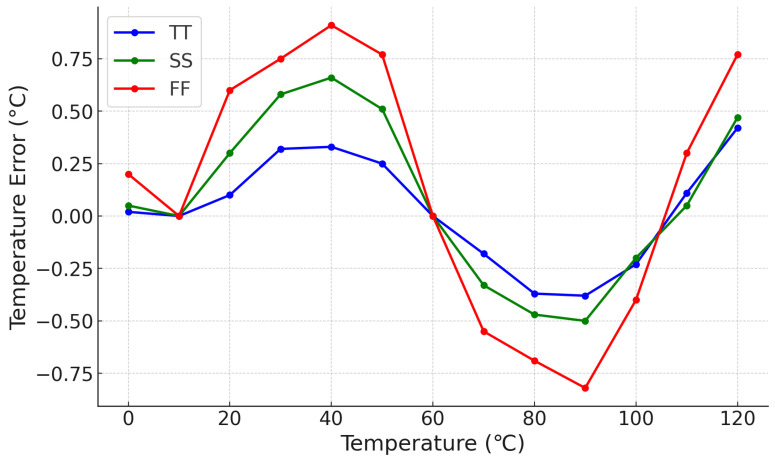
The simulated temperature error for different process corners.

Therefore, Figure 13 presents the simulated inaccuracy of the output temperature code within the target temperature detection range, considering different process corners and two-point calibration using 10 °C and 60 °C as temperature references. The absolute inaccuracy for the TT process corner is +0.43 °C/−0.38 °C. This result indicates that the proposed design has a low susceptibility to process variations.

**Figure 14:**
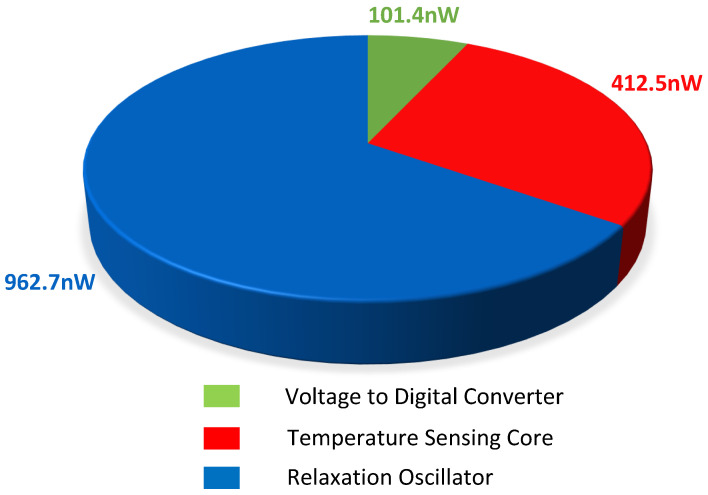
Power dissipation of the proposed temperature sensor.

The diode stacking structure of the temperature sensing core, the optimization of the comparator circuit’s static power consumption in the VTC, and the design of a comparator-free relaxation oscillator have resulted in a low-power temperature sensor. The total power consumption of the sensor is measured at 1.48 μW, with the relaxation oscillator accounting for a significant fraction of the total sensor power, specifically 962.7 nW. The power consumption of each part of the sensor is plotted in Figure 14.

The performance comparison of the proposed temperature sensor and several prior works is tabulated in Table 1. It must be stated that our results are based on simulations. Compared to other temperature sensors, the sensor proposed in this paper is featured with high accuracy and low power consumption, particularly excelling in resolution. The resolution of this sensor is better compared to sensors with the same readout structure.

It can be noted that the simulation is limited to the temperature range of 0∼120 °C to achieve the best performance in terms of inaccuracy and resolution. For a wider temperature range, the nonlinearity of the temperature sensing element becomes more significant, as the second-order derivative is always positive. It is scalable to a wider temperature range at the cost of resolution, depending on the application.

## 5. Conclusions

A CMOS-based temperature sensor with a time-domain readout is designed in a 180 nm CMOS process. It utilizes a reference current and voltage to bias the $V_{PTAT}$ and $V_{CTAT}$ generators, achieving high accuracy through a diode stacking structure that increases the TC of both $V_{PTAT}$ and $V_{CTAT}$. Additionally, a dual-input capacitor multiplexing VTC is used to enhance resolution by fully utilizing both temperature-negative and temperature-positive correlated information. Moreover, a low-temperature drift voltage-mode relaxation oscillator is included to effectively address the issue of large oscillation frequency drift caused by a significant temperature impact on the delay error. The simulation results show that the proposed temperature sensor has a 0.0071 °C resolution, a 1.9 pJ · K^2^ resolution FoM, an acceptable inaccuracy of +0.43 °C/−0.38 °C, and a power consumption of only 1.48 μW from 0 °C to 100 °C for 1.2 V supply after two-point calibration.

## Figures and Tables

**Table 1:** Performance comparison with other works.

Paper	JSSC [27]	TCAS-I [8]	TCAS-I [28]	TCAS-II [1]	TCAS-II [29]	MDPI [30]	This Work
Year	2022	2020	2021	2022	2023	2018	2024
Technology (nm)	110	180	130	65	28	180	180
Type	BJT	MOSFET	MOSFET	Res	Res	BJT	MOSFET
Supply Voltage (V)	1.35∼2	1.8	0.95	0.9	0.9	1.8	1.2
Temperature Range (°C)	−40∼140	−20∼80	0∼80	−5∼95	−40∼100	0∼100	0∼120
Resolution (°C)	0.144	0.1	0.1	0.0098	0.0565	0.01	0.0071
Conversion Time (ms)	0.8	25	59	10	0.404	3.4	26
Inaccuracy (°C)	+1/−1	+0.66/−0.73	+0.44/−0.4	+1.8/−1.6	+0.81/−0.62	+0.2/−0.2	+0.43/−0.38
Relative Inaccuracy ^1^ (%)	1.11	1.39	1.05	3.4	1.02	0.4	0.675
Power ^2^ (μW)	3.1	1.99	0.196	0.31	123.5	11.2	1.48
Energy/Conversion (nJ)	2.48	49.75	11.56	3.1	49.9	38	38.39
Resolution FOM ^3^ (nJ · K^2^)	0.0514	0.4975	0.1156	0.000297	0.159	0.0038	0.0019

^1^ Relative Inaccuracy (%) = Max error/Temperature range × 100. ^2^ Power or energy for generating external references not include. ^3^ Resolution FOM (nJ · K^2^) = Energy/Conversion × (Resolution)^2^.

## Data Availability

Data will be made available on request.
